# Introduction to the Special Issue—Nurturing resilient marine ecosystems

**DOI:** 10.1098/rstb.2021.0120

**Published:** 2022-07-04

**Authors:** Daniela N. Schmidt, Tayanah O'Donnell

**Affiliations:** ^1^ School of Earth Sciences, University of Bristol, Wills Memorial Building, Bristol BS8 1RJ, UK; ^2^ Australian National University, Canberra, Australia

**Keywords:** socio-ecological systems, cross-disciplinary, coast, climate change, commonwealth

## 1. Introduction

In February 2021, the (third) Commonwealth Science Conference (CSC) was co-organized by the Royal Society and the African Academy of Sciences. As 2021 was not like any other year, the meeting took place virtually from 22–26 February 2021. One of the aims of the CSC is to improve research links between outstanding early career researchers and scientists representing a range of disciplines and from across the Commonwealth to facilitate joint work on addressing global development challenges. The volume here draws on one of the three themed sessions: Nurturing resilient ecosystems, within which we explored maintaining and strengthening biodiversity, sustainable stewardship and adapting to a changing climate.

2021 also marked the beginning of the United Nations decade of Oceans Science for Sustainable Development. Most Commonwealth countries have a coastline and more than half of these are small island ‘big ocean’ nations with more ocean space than land space. This increased attention on marine spaces and their role in nurturing resilient ecosystems was evident in the range of contributions to the CSC resulting in the focus of this special issue.

Climate and biodiversity emergencies are being declared across the world based on an acknowledgement that the climate and biodiversity crisis can only be solved hand in hand [1]. The papers in this issue, therefore, were written against the backdrop of 2021 as a critical year to halt and reverse biodiversity loss and to address climate change impacts and adaptation, given the CO15 (biodiversity), COP26 (climate change) and the Commonwealth Heads of Government Meeting.

Nature and its ability to protect people against climate change impacts and help mitigating climate change has also been receiving increasing visibility at the COP26 in Glasgow [2]. The importance of nature in climate adaptation and mitigation is also a central point in the contribution of the Working Group II of the Intergovernmental Panel on Climate Change (IPCC) in the 6th Assessment Report [3]. The report states the growing risks for many unique threatened natural systems, such as coral reef ecosystems, cryosphere adapted and mountain ecosystems, and wetlands, with irreversible impacts projected for the near future for some of these systems. Further emphasis is given to the link between vulnerability of ecosystems to climate change and development of human society, such as unsustainable consumption, demographic pressures and non-climatic drivers such as habitat fragmentation, pollutants and unsustainable use [4]. Attention to the role of oceans in mitigation and the need for conservation is gaining widespread support and rapid momentum. However the IPCC report also clearly emphasizes the small area currently protected and insufficient stewardship to reduce damage or increase resilience [3].

The presentations at the meeting covered a wide range of topics and drew on knowledge and practice from a wide range of scientific backgrounds and methods. Our speakers came from all parts of the Commonwealth, many from Small Island Developing States (SIDS). The sessions were divided along three topics: trajectories, challenges and solutions for biodiversity; challenges and opportunities for the blue economy; and adaptation and mitigation challenges for ocean states in the era of climate change.

Dame Linda Partridge in opening the session focused on the human demands on nature which vastly outweigh nature's capacity to provide the goods and services we depend on [5]. She also introduced the Royal Society's own programme on how nature is valued and accounted for in decision-making; and cross-sectoral solutions to biodiversity loss, climate change and development. Breakout sessions were used to generate links between researchers, and consider how to strengthen capacity in light of global development challenges.

While the focus was on the ocean, the discourse drew on synergies between terrestrial and marine ecosystems and their challenges, for example in the context of restoration. The challenges can only be solved by addressing the interactions between environment, natural ecosystems and people. As such we are grateful to the editors of the Philosophical Transactions for supporting our inter- and cross-disciplinary approach to this special issue.

This volume comprises 12 articles, which are primary research articles, some reviews and some opinion pieces. Authors from around the Commonwealth report on studies in different regions highlighting the range of approaches and local experiences. As such it is not unexpected that the discussions during the meeting often came back to people and their reality of biodiversity loss and climate change impacts. The questions around impacts of climate change on nature, and the solutions natural systems can provide are wide ranging. The role of humans, institutions, and related systems in both framing and responding to these questions is wide-reaching, complex and value-laden. It is essential therefore that our scientific endeavours continue to pursue excellence in breadth and depth of diverse knowledge systems. The volume of contributions is trying to open the door to multiple ways of knowing, understanding and exploring the world including First Nations perspectives.

## 2. Trajectories, challenges and solutions for biodiversity

The volume starts with papers addressing critical ecosystem and iconic animals. Amon *et al*. in their article ‘*My deep sea, my backyard: a pilot study to* *expand global deep-ocean exploration’* [6] take us to the deep sea which includes greater than 90% of all habitable space in the ocean. The paper raises awareness of lack of current scientific knowledge, challenges for governance and inequitable global capacity for deep-ocean scientific exploration. Most areas of the deep ocean is under the stewardship of countries with developing economies. They are reporting on a project ‘My Deep Sea, My Backyard’ aimed to grow deep-ocean capacity in two countries, Trinidad and Tobago and Kiribati by using low-cost technology while building lasting in-country capacity.

Kebke *et al*. in their contribution ‘*Climate change and cetacean health: impacts and future directions*' [7] provide an overview of the climate change impacts the foraging opportunities, which combined with habitat loss, forces cetaceans to move to new feeding grounds. Increased Arctic meltwater and rainfall events are projected to lead to higher rates of land-based runoff in downstream coastal areas. These combined anthropogenic stressors threaten taxa with low population numbers or those with a limited habitat range. Persistent and mobile contaminants bioaccumulate in the ecosystem, with potentially severe consequences for reproduction, health and metabolism of marine mammals.

Tulloch *et al*.'s paper ‘*Accounting for indirect cumulative effects of human activities for salmon-linked land and ocean ecosystems*' [8] similarly focuses on the link between changes on land and the ocean with the examples of the effects of human-driven pressures in salmon- and herring-linked ecosystems of western Canada. They clearly highlight the importance of taking indirect risks for species on land and the ocean into consideration. Using this framework resulted in the greatest change in risk for low trophic marine species and increased the cumulative risk for salmon and herring. The framework can inform immediate management of linked land–sea ecosystems.

## 3. Blue Economy and nature-based solutions

The issue then continues with several papers showing the interconnections of people and nature, especially around opportunities for the Blue Economy and Nature-based solutions (NbS) [9–12]. Natural and seminatural ecosystems can make fundamental contributions to climate change mitigation, livelihoods, and protecting people from climate change impacts [9]. A recent stocktake identified clear gaps in our knowledge of adaptation options which included: the effectiveness of adaptation responses, limits to adaptation, enabling adaptation in missing places, providing scholars and scholarship, synthesizing different forms of evidence [13], and the following papers give some examples.

Voyer *et al*. introduce '*The Blue Economy in the Commonwealth: variations and consistencies'* [14]. The Blue Economy describes a wide variety of development approaches and priorities in ocean and coastal areas. They explore what Blue Economy means in different settings, governance strategies and implementation approaches by analysing key policy statements and governance instruments within the context of Sustainable Development Goals (SDGs) and the Commonwealth Blue Charter. They assess the balance between economic and environmental objectives and equity objectives including food security and gender equality. The Blue Economy may be facilitating integration across sectoral management, with the emergence of boundary-crossing arrangements.

One approach of the Blue Economy is discussed in Alleway *et al.*'s ‘*Leveraging global and domestic opportunities to grow climate-smart mariculture*’ [15]. The paper assessed the mariculture food system in 171 coastal countries for vulnerability to climate change and opportunities to deliver climate mitigation. They identified higher immediate opportunity for adaptation and mitigation in Northern America and Europe. However, even in regions with lower vulnerability, vulnerabilities and opportunities are place dependent and vary within and between all regions and countries, largely owing to the existing mariculture systems, human development and governance capacity. They highlight solutions need to reflect local practice, needs and constraints.

In some systems though, the need for adaptation for nature and people is becoming vital as adaptive capacities are reached and projected to be exceeded in the near future. One of these are small island—large ocean states. Barnett *et al*. explore the potential for '*Nature-based solutions for atoll habitability'* [16], drawing on these closely co-dependent environmental and social systems. NbS in the context of atolls peoples though are hindered as knowledge of their application, potential and enablers, barriers and limits is fragmented. NbS could make a major contribution to sustain the habitability of atolls through a changing climate, though their success will be a function of not just their ecological performance but also their fit with atoll customs and institutions. They outline a systematic and transdisciplinary agenda to better understand the enablers, barriers, and limits to NbS for atoll habitability.

## 4. Enablers of a resilient marine socio-ecological system

The last suite of papers assesses approaches to address challenges of the climate and ecological emergencies which play out acutely in ocean systems with devastating impacts on marine biodiversity, and livelihoods of coastal communities and their cultural values. People are dependent on our oceans for economic, health, and social benefits but oceans need to be protected to deliver on these services, conserving and restoring their diversity as aimed for in the SDG 14, by 2030.

Kristy de Salas *et al*. present their view on ‘*The super wicked problem of ocean health: a socio-ecological perspective’* [17]. They argue that the changes needed to prevent further ocean ecosystem degradation or limit the impact of existing degradation, are not being undertaken. Using a socio-ecological lens they explore the nature of the actors and behaviours for change at the local, community, state, national and international levels. They emphasize the need for technology, information and knowledge sharing, and to address the challenges of ocean health and promote resilience with transformational teams and leaders. Transformative polices within a holistic and integrated system ensure ocean health initiatives are pathways to change.

Schmidt *et al*. assess marine protected areas (MPAs) as a management tool to address the question ‘*Are coasts socio-ecological systems with benefits for humans and nature*’ [18]. They argue that MPAs can strengthen bio-cultural diversity and sustainability of coastal social-ecological systems. MPA governance must be cognizant of the interdependency between natural and human systems and their reaction to climate change based on an integrated, co-developed, and interdisciplinary approach. They question whether MPAs are ecologically effective while facing key climate and regulatory uncertainties and questions of legitimacy. Focusing on the UK as a case study, they highlight some of the challenges to achieve effective, adaptive and legitimate governance of MPAs.

Richter *et al*. discuss the importance of research across disciplines by ‘*Integrating knowledge systems: connecting natural and social science data to understand* *temporal trends of marine ecosystem quality****‘*** [19]*.* They argue that successful resource management relies on natural science and social science data to underpin feasible and effective policies and management interventions. They propose a methodology to assess temporal trends in ecosystem quality based on systematic mapping. Combining time series datasets of populations of fisheries stocks, satellite-derived habitat maps with people's perceptions complements the information and reduces the shortcomings. They discuss the limitations of the suggested approach and potential implications for resource management and communication strategies.

O'Donnell in ‘*Managed retreat and planned retreat: a systematic literature review*’ [20] is demonstrating a four-fold increase of published papers discussing retreat from 2012–2016 in the Web of Science database. Trends are discussed with respect to governance and institutional responses, of private property (materially and in discourse), and social and environmental justice issues including: equity, race, health and wellbeing and housing accessibility. A range of terminology to describe managed retreat or planned retreat was captured. Analysis posits that managed retreat ought to be the preferred frame so that scholarly literature can be better aggregated. In addition, managed retreat ought to refer to retreat after an event, and planned retreat to retreat implemented before an event.

This group of papers and the discussion at the CSC leaves the question of what the role of the Commonwealth is in joint action to mitigate climate change and reduce the risks of future warming on people, ecosystems and their services. During the meetings, coordinated data sharing and long-term monitoring of biodiversity across the Commonwealth were considered as one achievable goal. Other suggestions included databases for impacts on ecological systems, information on adaptation options that work, in which context and for whom, the sharing of novel technological approaches to quantify impacts, supported by training for early career researchers. In addition, recognition of the diverse range and rate of climate impacts across the Commonwealth, and for global south countries in particular, was discussed at length with the role of other Commonwealth countries to support adaptation efforts emphasized. For most SIDS, there are specific needs driven by the urgency of the near term risks which warrant close attention. The long-standing relationships of the Commonwealth Heads of Government is an asset in global coordination of governance across boundaries to move from pledges to small steps today for more actions in the next decades.

## Data Availability

This article has no additional data.
